# Transfusion Burden in Neonates with Hypoxic–Ischemic Encephalopathy Undergoing Therapeutic Hypothermia

**DOI:** 10.3390/children13091185

**Published:** 2026-09-02

**Authors:** Domenico Umberto De Rose, Chiara Maddaloni, Sara Ronci, Francesca Campi, Stefano Caoci, Ludovica Martini, Iliana Bersani, Immacolata Savarese, Irma Capolupo, Daniela Longo, Pierpaolo Berti, Ottavia Porzio, Matteo Luciani, Andrea Dotta

**Affiliations:** 1 Neonatal Intensive Care Unit, “Bambino Gesù” Children’s Hospital IRCCS, 00165 Rome, Italy; domenico.derose@opbg.net (D.U.D.R.); chiara.maddaloni@opbg.net (C.M.); francesca.campi@opbg.net (F.C.); stefano.caoci@opbg.net (S.C.); ludovica.martini@opbg.net (L.M.); immacolata.savarese@opbg.net (I.S.); irma.capolupo@opbg.net (I.C.); andrea.dotta@opbg.net (A.D.); 2 Neonatal Semi-Intensive Care and Follow-Up Unit, “Bambino Gesù” Children’s Hospital IRCCS, 00165 Rome, Italy; iliana.bersani@opbg.net; 3 Functional and Interventional Neuroradiology Unit, “Bambino Gesù” Children’s Hospital IRCCS, 00165 Rome, Italy; daniela.longo@opbg.net; 4 Transfusion Medicine Unit, “Bambino Gesù” Children’s Hospital IRCCS, 00165 Rome, Italy; pierpaolo.berti@opbg.net (P.B.); ottavia.porzio@opbg.net (O.P.); 5 Department of Experimental Medicine, “Tor Vergata” University, 00133 Rome, Italy; 6 Pediatric Hematology and Oncology Unit, “Bambino Gesù” Children’s Hospital IRCCS, 00165 Rome, Italy; matteo.luciani@opbg.net

**Keywords:** perinatal asphyxia, neonatal intensive care, blood product exposure, transfusion thresholds, blood products, fresh frozen plasma, platelet transfusion, red blood cell transfusion, prothrombin complex concentrate

## Abstract

**Highlights:**

**What are the main findings?**

- Over half (52.1%) of neonates with HIE undergoing therapeutic hypothermia received at least one blood component and coagulation/plasma-derived products, with a median of 2 transfusion episodes per infant; plasma and prothrombin complex concentrate were most frequently used.
- Clinically visible bleeding (aOR 12.95) and need for respiratory support (aOR 2.98) were independently associated with transfusion exposure, and transfused infants more often showed pathological brain MRI findings.

**What are the implications of the main findings?**

- Transfusion burden in this population appears to reflect underlying disease severity rather than acting as an independent driver of neurological injury.
- These findings support the development of evidence-based, severity-adjusted transfusion strategies to optimize blood product stewardship in neonates with HIE.

**Abstract:**

**Background/Objectives**: Neonates with hypoxic–ischemic encephalopathy (HIE) undergoing therapeutic hypothermia (TH) frequently develop coagulation abnormalities and bleeding complications, yet data on transfusion burden and its clinical correlates in this population remain limited. **Materials and Methods**: We performed a secondary analysis of a retrospective single-center cohort of neonates with HIE treated with TH between 2014 and 2022. Transfusion burden was defined as receipt of at least one blood component and coagulation/plasma products during hospitalization and the total number of transfusion episodes. Demographic, perinatal, biochemical, and clinical severity variables were collected. Univariable and multivariable logistic regression analyses were used to identify factors independently associated with transfusion exposure. Brain magnetic resonance imaging (MRI) findings were compared between transfused and non-transfused infants. **Results**: Among 142 included neonates, 74 (52.1%) received at least one blood product. The median number of transfusion episodes among transfused infants was 2 (IQR 1–3). Fresh frozen plasma and prothrombin complex concentrate were the most frequently administered products. Transfused infants showed higher markers of illness severity. In multivariable analysis, clinically visible bleeding (adjusted odds ratio [aOR] 12.95, 95% CI 1.34–124.97) and need for respiratory support (aOR 2.98, 95% CI 1.19–7.45) remained independently associated with transfusion exposure. Pathological brain MRI findings, including intracranial bleeding and hypoxic–ischemic injury, were more frequent among transfused infants. **Conclusions**: blood components and coagulation/plasma-derived products transfusions are common in neonates with HIE undergoing TH and appear to primarily reflect underlying disease severity. These findings highlight the need for optimized, evidence-based transfusion strategies in this vulnerable population.

## 1. Introduction

Hypoxic–ischemic encephalopathy (HIE) continues to be a major cause of death and disability among term and near-term newborns. Therapeutic hypothermia (TH) is the established standard of care for neonates with moderate to severe HIE and has been consistently shown to reduce death and disability when initiated within the first 6 h of life [1,2].

Despite its neuroprotective benefits, TH is associated with systemic effects, including cardiovascular instability, metabolic derangements, and alterations of the coagulation system. Perinatal asphyxia disrupts neonatal hemostasis through multiple mechanisms, including impaired hepatic synthesis of coagulation factors, platelet dysfunction, endothelial injury, and activation of inflammatory and consumptive pathways [3,4,5]. These abnormalities may be further exacerbated by TH, which lowers coagulation enzyme activity and reduces platelet function, raising bleeding risk and the need for blood components and coagulation/plasma-derived product transfusions [6].

Coagulation disturbances and bleeding complications have been increasingly recognized as clinically relevant issues in neonates undergoing TH, yet evidence guiding transfusion practices in this setting remains limited. In particular, transfusion thresholds for red blood cells, plasma, platelets, and adjunctive coagulation/plasma-derived products are largely extrapolated from general neonatal populations and are often based on expert opinion [7,8].

In a recent study, our group characterized coagulation profiles and reference percentiles in neonates with HIE treated with TH, demonstrating marked alterations in conventional coagulation parameters during the early phases of treatment and highlighting the complexity of hemostatic management in this population [9].

Blood components and coagulation/plasma-derived products transfusion is a frequent intervention in critically ill neonates, and in HIE it may act less as a therapeutic measure and more as a marker of underlying disease severity, reflecting the combined effects of systemic illness, bleeding complications, and neurological injury. In neonates with moderate to severe HIE, approximately 69% exhibit hemostatic dysfunction, with 28% experiencing abnormal bleeding and 57% requiring at least one blood product transfusion [10].

A retrospective study of care practices for infants with HIE treated with TH across 24 Canadian tertiary NICUs within the Canadian Neonatal Network from 2010 to 2020 found considerable variability in the proportion of infants receiving blood products between centers, ranging from 9 to 21% [11] and similar to the Canadian experience, European data from the TOBY register highlighted that up to 41% and 58% of cooled infants in the TOBY trial presented with prolonged coagulation time and thrombocytopenia, respectively, although the transfusion burden was not reported [12]. Similarly, up to 14.5% and 25.8% of cooled infants from the neo.nEURO.network RCT had coagulopathy and thrombocytopenia, respectively, although transfusion burden was not reported [13]. Understanding transfusion burden and its association with clinical severity indicators and neuroimaging findings is therefore essential to improve blood stewardship.

In this study, we aimed to quantify transfusion burden during hospitalization in HIE infants and identify clinical and biochemical factors independently associated with receipt of at least one blood product. In addition, we explored the relationship between transfusion exposure and brain magnetic resonance imaging (MRI) findings, hypothesizing that transfusion exposure may serve as a proxy for underlying disease severity rather than a direct causal determinant of neurological injury.

## 2. Materials and Methods

### 2.1. Study Design and Data Collection

This study is a secondary analysis of a previously described retrospective cohort of neonates with HIE undergoing TH at a single tertiary-level Neonatal Intensive Care Unit with a distinct primary objective focused on transfusion burden and factors associated with transfusion exposure. The original cohort included all consecutive neonates admitted between November 2014 and March 2022 who fulfilled national criteria for TH [9].

For the present analysis, all infants from the original cohort with complete data on transfusion exposure during hospitalization were included. Transfusion exposure was assessed over the entire hospital stay, from admission to discharge, and was not limited to the period of therapeutic hypothermia. All infants were outborn, and admission laboratory examinations were obtained in most cases still on passive hypothermia (core temperature of 35–36 °C), during the process of initiating active cooling: only in a few infants coming from far hospitals was the active cooling process already started during the transportation, to begin TH within 6 h of birth. All infants underwent blood chemistry exams, including a full coagulation profile, at hospital admission within 6 h of life (T0), and then at 24 h (T1), 48 h (T2), 72 h (T3), and after rewarming (T4). As all infants were outborn, baseline (T0) sampling was obtained in most cases while still in passive hypothermia (core temperature 35–36 °C), during initiation of active cooling, and therefore largely reflects the coagulation status related to perinatal asphyxia/HIE itself rather than an effect of therapeutic hypothermia per se. We acknowledge that this design does not allow definitive separation of HIE-related coagulopathy from TH-induced alterations, since even baseline sampling occurred during the early cooling process.

The transfusion thresholds and clinical criteria described in the paper remained unchanged in our Neonatal Intensive Care Unit throughout the study period, and they were part of our institutional protocol for the management of neonates with HIE undergoing TH.

Clinical and laboratory data were retrieved retrospectively from hospital records and the local transfusion registry. The following variables were collected: demographic and perinatal data (gestational age, birthweight, sex, Apgar scores, worst pH, base excess, and lactate at birth) and clinical severity indicators (Sarnat stage, need for respiratory support, occurrence of seizures, early exit from TH). For transfusion data, we collected: exposure to any blood product, type of administered blood component (red blood cells, fresh frozen plasma, and platelets) and coagulation/plasma-derived product (prothrombin complex concentrate, antithrombin, fibrinogen concentrate), number of transfusion episodes per patient, and cumulative number of different blood components and coagulation/plasma-derived products received per patient. A transfusion episode was defined as each distinct administration of a blood component or coagulation product, counted separately even when different products were administered on the same calendar day.

Brain magnetic resonance imaging (MRI) was performed at about 7 days of life, following completion of TH, and was available for all included infants. Images were independently reviewed by two pediatric neuroradiologists blinded to transfusion status, with discordant readings resolved by a third senior neuroradiologist. Intracranial bleeding was defined as blood products in intra- or extra-axial compartments on susceptibility-weighted/T1-weighted sequences; hypoxic–ischemic injury was defined as abnormal signal intensity and/or restricted diffusion in regions typically affected by perinatal asphyxia (basal ganglia, thalami, perirolandic cortex, white matter).

### 2.2. Blood Transfusion Thresholds

In our unit, we transfused all HIE patients with irradiated, leukocyte-depleted red blood cells when their hemoglobin is below 10 g/dL or below 11 g/dL if requiring respiratory support. We administered fresh frozen plasma (FFP) in patients with prothrombin time expressed as International normalized ratio (PT-INR) > 2. FFP is also administered in cases with lower values of PT-INR (1.50–1.99) and low fibrinogen (<150 mg/dL). Conversely, infants with PT-INR 1.50–1.99 and normal fibrinogen received a prothrombin complex concentrate (PCC) (Confidex^®^, CSL Behring, Marburg, Germany), whereas infants with normal PT-INR values but fibrinogen < 150 mg/dL underwent cryoprecipitate. We transfused with irradiated, leukocyte-depleted platelets all patients with a platelet count < 25 × 10^9^/L and patients with major bleeding and 25–49 × 10^9^ platelets/L. Antithrombin deficiency was usually corrected for infants with antithrombin ≤ 60%. The transfusion thresholds described below represent a formally predefined institutional threshold unchanged during the study period.

Clinical discretion was possible for all borderline cases or those with any clinically visible bleeding (such as pulmonary bleeding, hematuria, umbilical bleeding, nasal bleeding, gastric bleeding, or petechiae).

### 2.3. Study Objectives and Outcomes

The primary outcomes were the transfusion burden, defined as the proportion of HIE infants receiving at least one blood component or coagulation product during hospitalization, and the total number of transfusion episodes per patient.

Secondary outcomes included the distribution of different blood components and coagulation/plasma-derived products administered, cumulative exposure to multiple blood product types, comparison between transfused and not-transfused infants, and identification of clinical and biochemical factors associated with increased transfusion exposure. Given the clinical heterogeneity of these products, transfusion exposure was also examined separately for blood components and for coagulation/plasma-derived products as a sensitivity analysis.

### 2.4. Statistical Analysis

Categorical variables are presented as numbers and percentages. Continuous variables are expressed as mean ± standard deviation or median and interquartile range (IQR), as appropriate. Comparisons between transfused and non-transfused infants were performed using the chi-square test or Fisher’s exact test for categorical variables, and Student’s *t*-test or Mann–Whitney U test for continuous variables.

To explore factors independently associated with transfusion exposure, univariable analyses were initially performed. Variables significantly associated with transfusion exposure (*p* < 0.05) in univariable analysis were entered simultaneously into a multivariable logistic regression model, without a stepwise selection procedure.

A *p*-value < 0.05 was considered statistically significant. Statistical analysis was performed using the software Jamovi (version 2.7.16 for macOS, Sidney, Australia).

## 3. Results

### 3.1. Study Population

During the study period, 145 infants met TH criteria; 142 with complete transfusion data were retained for analysis. Baseline demographic and clinical characteristics of the study population are summarized in Table 1. Among the included infants, none died during the study period.

### 3.2. Transfusion Burden

Overall, 74 (52.1%) infants received at least one blood component or coagulation product during hospital stay, whereas 68 (47.9%) did not receive any transfusion. Among transfused infants, the total number of transfusion episodes was 215, with a median of 2 (IQR 1–3) episodes per infant. In the majority of cases, transfusion occurred on the first day of life. During hospital stay, 48/142 (33.8%) infants were exposed to a single blood component or coagulation product, and 26/142 (18.3%) received two or more different blood components or coagulation products. The frequency of administration is detailed in Table 2: fresh frozen plasma and prothrombin complex concentrate were the most frequently used products in this cohort.

**Figure 1:**
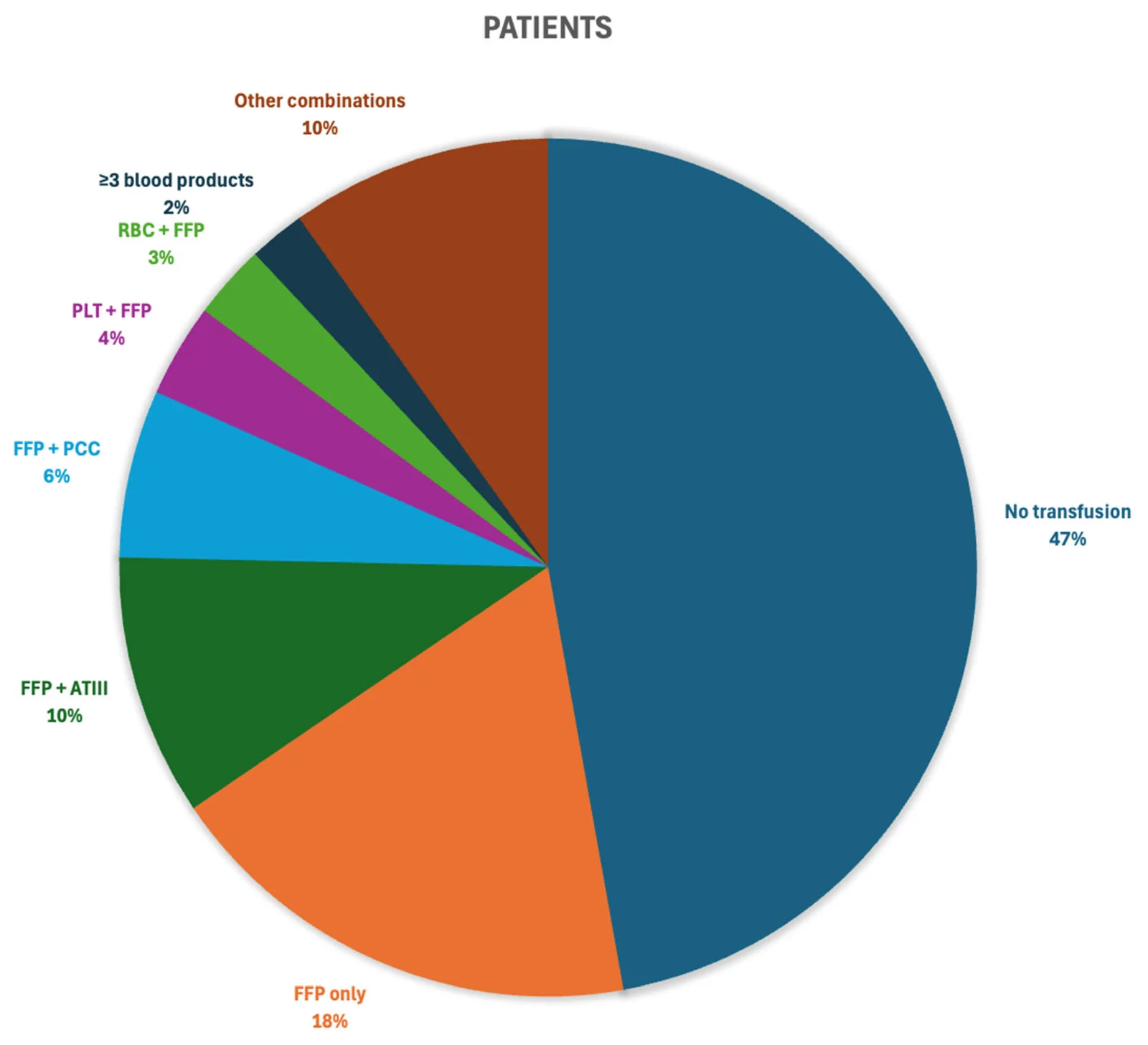
Distribution of transfusion exposure in the study cohort.

Figure 1 illustrates the proportion of infants who did not receive any transfusion and the relative contribution of different products and their combinations among transfused infants.

### 3.3. Comparison Between Transfused and Not-Transfused Infants

Comparisons between transfused and non-transfused infants are presented in Table 1. Infants who received at least one transfusion more frequently required respiratory support during therapeutic hypothermia and showed higher markers of illness severity at birth, including lower 1st minute and 5th minute Apgar scores, more severe metabolic acidosis, and elevated lactate levels.

Clinically visible bleeding was strongly associated with transfusion exposure, as 15/16 (93.8%) infants with bleeding received transfusions compared with 59/126 (46.8%) infants without bleeding (χ^2^ = 12.5, *p* < 0.001).

### 3.4. Factors Associated with Transfusion Exposure

In univariable analyses, cesarean section, worse metabolic acidosis (base excess), higher lactate levels at birth, clinically visible bleeding, need for respiratory support, and electroencephalographic seizures were associated with receipt of at least one blood product. In multivariable logistic regression analysis, clinically visible bleeding (aOR 12.95, 95% CI 1.34–124.97; *p* = 0.027) and need for respiratory support (aOR 2.98, 95% CI 1.19–7.45; *p* = 0.020) remained independently associated with transfusion exposure, whereas cesarean section, metabolic acidosis, lactate levels, and EEG seizures were no longer significant after adjustment (Table 3).

The distribution of brain MRI findings differed significantly between transfused and non-transfused infants (χ^2^ = 7.19, *p* = 0.027). Notably, intracranial bleeding and hypoxic–ischemic injury on brain MRI were more frequently observed among infants who received transfusions, likely reflecting a higher underlying disease severity, whereas a normal MRI was more common in non-transfused infants (Table 4).

## 4. Discussion

Blood components or coagulation product transfusions are common in neonates with HIE undergoing therapeutic hypothermia, but evidence guiding transfusion thresholds and neurodevelopmental implications remains limited.

In this study, we describe transfusion practices at a high-volume level III NICU and investigate whether transfusion exposure is a marker of underlying disease severity rather than an independent driver of neurological injury.

In our cohort of neonates with hypoxic–ischemic encephalopathy undergoing TH, a high proportion of infants received at least one blood component or coagulation product during hospitalization, with a median of two transfusion episodes per transfused infant. FFP and PCC accounted for the largest share of transfusions administered. Our findings of a high transfusion burden are consistent with previous observational data indicating frequent hemostatic dysfunction and bleeding in HIE, where hemostatic abnormalities were present in approximately two-thirds of infants and over half required at least one blood component or coagulation product transfusion [10].

This is consistent with recent retrospective data on hemostatic management in HIE infants treated with TH [9,14] that explore how neonates with HIE commonly experience coagulation abnormalities, driven by systemic oxygen deprivation that disrupts multiple components of hemostasis including impaired synthesis of coagulation factors, platelet dysfunction, and endothelial injury [3].

Traditional coagulation tests such as prolonged PT/INR, elevated aPTT, low fibrinogen, and thrombocytopenia are universal in this population, but their predictive value for clinical bleeding remains unclear, contributing to considerable variability in transfusion decision-making [9]. TH further alters coagulation by reducing enzymatic activity within the coagulation cascade and impairing platelet function, which may increase bleeding risk and influence clinicians toward more liberal use of blood products, even in the absence of strong outcome data supporting benefit [3].

Blood products are administered to improve oxygen delivery and support hemodynamics, but transfusions also stimulate systemic inflammation, pro-inflammatory cytokine release, and endothelial activation, which have been implicated in adverse outcomes in critically ill neonates [15,16].

Blood product transfusion in neonates is not without risk, and potential adverse effects (including transfusion-associated circulatory overload, transfusion-related immunomodulation, allergic or febrile reactions, and infectious risk) should be carefully weighed against the clinical benefit of transfusion in this vulnerable population. Current evidence in neonatology supports a shift toward more restrictive transfusion thresholds in neonatal populations, such as restrictive platelet transfusion thresholds have been shown to reduce the number of platelet transfusions and may be safe without increasing major hemorrhage, with some evidence of potential long-term benefit in very preterm infants [17].

In extremely low birth weight infants, using higher hemoglobin cutoffs increased transfusion frequency but did not improve discharge survival or morbidity outcomes. According to the PINT trial, no significant differences were observed between low and high threshold groups in primary or secondary outcomes [18].

Liberal red blood cell transfusion practices in preterm infants have not been linked to increased mortality or significant neurodevelopmental impairment at 24 months of age [19,20].

A recent online survey involving 608 NICUs documented that blood transfusion practices vary by country and within networks. Two-thirds of NICUs have implemented a limited transfusion protocol based on current recommendations [21].

Blood product transfusion has been studied extensively in sepsis and prematurity, but data on transfusion practice in HIE and its association with clinically significant outcomes remain limited, and no uniform transfusion guideline exists for this population. Consistent with these observations, recent single-center HIE cohort data show that univariable analyses can link transfusion exposure to an increased in-hospital mortality, but multivariable adjustment for disease severity often attenuates this association, suggesting higher transfusion rates track illness severity rather than causing worse outcomes [14].

In our cohort, clinically evident bleeding was strongly associated with transfusion exposure, with nearly all infants with bleeding receiving transfusions compared with fewer than half without bleeding. Transfused infants also showed higher illness severity overall: lower Apgar scores, more pronounced metabolic acidosis, higher lactate, and greater need for respiratory support and intracranial bleeding and hypoxic–ischemic injury on brain MRI. In particular, the comparison of pathological brain MRI findings between transfused and not-transfused infants was unadjusted, and the higher frequency of pathological MRI findings among transfused infants likely reflects confounding by underlying disease severity rather than a direct effect of transfusion on neurological injury; this association should not be interpreted as causal.

This study has several limitations. First, its retrospective design inherently limits causal inference and is subject to residual confounding. Although multivariable analyses were performed to adjust for relevant clinical and biochemical covariates, unmeasured factors related to illness severity or clinician decision-making may have influenced transfusion practices. Second, this is a single-center study, which may limit the generalizability of our findings. Transfusion thresholds and clinical management of coagulation abnormalities during therapeutic hypothermia are not standardized across centers and may vary according to local protocols and clinician experience. Additionally, because baseline coagulation testing was performed during early passive/active cooling, this study cannot definitively distinguish coagulopathy attributable to perinatal hypoxic–ischemic injury from that induced or exacerbated by therapeutic hypothermia itself. Third, transfusion exposure in this cohort likely reflects confounding by indication. Blood product administration was driven by a combination of laboratory abnormalities, clinically visible bleeding, and overall clinical severity. Transfusion exposure in this cohort should therefore be interpreted as a proxy of disease severity, not an independent causal factor. Relatedly, the comparison of brain MRI findings between transfused and non-transfused infants was unadjusted for illness severity. The higher frequency of pathological MRI findings in transfused infants may similarly reflect underlying severity rather than a direct effect of transfusion itself. Fourth, the number of infants with clinically visible bleeding and specific bleeding subtypes was relatively small, resulting in wide confidence intervals for some effect estimates. This limited statistical power may have reduced our ability to detect independent associations for certain variables in multivariable models. Finally, long-term neurodevelopmental outcomes were not available for this analysis. As a result, the clinical implications of transfusion burden on longer-term outcomes could not be assessed and warrant further investigation in prospective, multicenter studies. Future research should focus on prospective, multicenter studies and randomized controlled trials to define safe and effective transfusion thresholds specific for HIE neonates undergoing TH. Integrating point-of-care hemostatic monitoring with standardized clinical criteria could support individualized transfusion strategies that limit unnecessary blood product exposure without compromising neurological outcomes.

## 5. Conclusions

Blood component or coagulation product transfusions were frequent in neonates with HIE undergoing therapeutic hypothermia and appeared to primarily reflect underlying disease severity. Clinically visible bleeding and need for respiratory support were independently associated with transfusion exposure, while transfused infants showed more severe neuroimaging abnormalities. However, further prospective studies are needed to optimize transfusion strategies in this population and define whether using optimal transfusion thresholds could impact clinical and neurodevelopmental outcomes.

## Figures and Tables

**Table 1:** Clinical and demographic characteristics of the study population.

	Total (n = 142)	Transfused Infants (n = 74)	Not-Transfused Infants (n = 68)	*p*-Value
Males, *n* (%)	80 (56.4%)	38 (26.8%)	42 (29.6%)	0.149
Birthweight (g)	3207 ± 529	3180 ± 581	3237 ± 468	0.152
Gestational age (weeks)	39 (38–40)	39 (38–40)	40 (38–40)	0.401
1st-minute Apgar score	4 (2–5)	3 (2–5)	4 (3–6)	**0.001**
5th-minute Apgar score	7 (5–7)	6 (4–7)	7 (6–8)	**0.006**
Moderate encephalopathy according to Sarnat staging, *n* (%)	129 (90.8%)	65 (45.8%)	64 (45.1%)	0.195
Severe encephalopathy according to Sarnat staging, *n* (%)	13 (9.2%)	9 (6.3%)	4 (2.8%)	
Worst pH at birth	7.02 ± 0.15	7.01 ± 0.15	7.05 ± 0.13	0.133
Worst base excess at birth (mmol/L)	−16.5 ± 5.3	−17.6 ± 5.2	−15.3 ± 5.2	**0.013**
Worst lactates at birth (mmol/L)	11.1 ± 4.3	12.3 ± 4.8	9.6 ± 3.1	**0.001**
Need for respiratory support, *n* (%)	75 (52.8%)	51 (35.9%)	24 (16.9%)	**<0.001**
Early exit from therapeutic hypothermia (TH), *n* (%)	5 (3.5%)	5	0	0.059
Electroencephalographic (EEG) seizure, *n* (%)	36 (25.4%)	25 (17.6%)	11 (7.7%)	**0.016**

**Table 2:** Distribution of blood product transfusions among study infants.

Blood Components	Infants Exposed, *n* (%)
Red blood cells	15 (10.6%)
Platelets	14 (9.9%)
Plasma (FFP)	55 (38.7%)
**Coagulation/Plasma-Derived Products**	**Infants Exposed, *n* (%)**
Prothrombin complex concentrate (PCC)	29 (20.4%)
Fibrinogen	1 (0.7%)
Antithrombin (ATIII)	20 (14.1%)

Percentages do not sum to 100%, as infants may have received more than one blood component type during hospitalization.

**Table 3:** Factors associated with receipt of at least one blood product.

Variable	Univariable OR (95% CI)	*p*-Value	Multivariable aOR (95% CI)	*p*-Value
Gestational age	0.86 (0.71–1.04)	0.114	-	
Sex	0.61 (0.31–1.20)	0.611	-	
Cesarean section	2.09 (1.06–4.10)	**0.033**	1.34 (0.55–3.30)	0.519
Worst pH at birth	0.15 (0.01–1.78)	0.135	-	
Worst base excess at birth (per 1 mmol/L decrease)	0.92 (0.85–0.98)	**0.016**	0.91 (0.81–1.02)	0.095
Worst lactates at birth (per 1 mmol/L increase)	1.17 (1.06–1.30)	**0.002**	1.11 (0.97–1.27)	0.137
Clinically visible bleeding	16.5 (2.12–128.71)	**0.007**	12.95 (1.34–124.97)	**0.027**
Need for respiratory support	4.32 (2.14–8.74)	**<0.001**	2.98 (1.19–7.48)	**0.020**
Electroencephalographic (EEG) seizure	2.54 (1.14–5.69)	**0.023**	1.24 (0.42–3.64)	0.700

**Table 4:** Transfusion exposure and pathological brain MRI findings.

Brain MRI Findings	Transfused Infants (*n* = 74)	Not Transfused Infants (*n* = 68)	Total (*n* = 142)
Normal MRI	37 (50.0%)	46 (67.6%)	83 (58.5%)
Intracranial bleeding	16 (21.6%)	15 (22.1%)	31 (21.8%)
Hypoxic–ischemic injury (HIE)	21 (28.4%)	7 (10.3%)	28 (19.7%)

## Data Availability

After the publication of the manuscript, formal requests for study data should be made to the corresponding author using a request form delineating research aims, methods, and the variables needed. If research questions and methods are considered relevant and valid, we will securely transfer the requested, fully anonymized data in the desired format to the party under data transfer agreements. After discussing this with the interested party, the team will decide about co-authorships. Data requests can be submitted at any time, and the data will be accessible for 12 months from publication, with possible extensions considered.

## Abbreviations

The following abbreviations are used in this manuscript:

ATIII	Antithrombin III
aOR	Adjusted odds ratio
aPTT	Activated partial thromboplastin time
CI	Confidence interval
EEG	Electroencephalographic
FFP	Fresh frozen plasma
HIE	Hypoxic–ischemic encephalopathy
IQR	Interquartile range
MRI	Magnetic resonance imaging
NICU	Neonatal intensive care unit
PCC	Prothrombin complex concentrate
PINT	Premature Infants in Need of Transfusion (trial)
PT-INR	Prothrombin time–international normalized ratio
RCT	Randomized controlled trial
TH	Therapeutic hypothermia
TOBY	Total Body Hypothermia for Neonatal Encephalopathy (trial)
